# Stress Alleviation Methods for community-Based Health ActiVists (SAMBHAV): Development of a digital program for stress reduction for community health workers in rural India

**DOI:** 10.1016/j.ssmmh.2023.100230

**Published:** 2023-06-17

**Authors:** Ritu Shrivastava, Abhishek Singh, Azaz Khan, Shivangi Choubey, Juliana Restivo Haney, Eirini Karyotaki, Deepak Tugnawat, Anant Bhan, John A. Naslund

**Affiliations:** a Sangath, Bhopal, India; b Department of Psychology, West Virginia University, Morgantown, WV, USA; c Department of Clinical, Neuro- and Developmental Psychology, Vrije Universiteit Amsterdam, Amsterdam, Netherlands; d Department of Global Health and Social Medicine, Harvard Medical School, Boston, MA, USA

**Keywords:** Community health worker, ASHA, Stress, Burnout, Digital technology, Mental health, Self-help plus

## Abstract

**Background:**

Community health workers (CHWs) face high levels of stress (both professional and personal) and risk of burnout, highlighting the need for efforts to promote their mental health and well-being. This study seeks to develop a digital stress reduction program for CHWs in rural India.

**Methods:**

A stepwise design process was employed to adapt and digitize the evidence-based World Health Organization’s Self-Help Plus (SH+) intervention for addressing psychological distress among CHWs in Madhya Pradesh, India. This involved participatory design workshops with CHWs to ensure that the digital stress reduction intervention would be relevant for their needs and the local culture and context. Small groups of CHWs reviewed the adapted program content, allowing the research team to make refinements such as simplifying language, tailoring content to the local setting, and ensuring that program materials are both interesting and relevant. Simultaneously, the research team digitized the content, leveraging a combination of video and graphical content, and uploaded it to the Sangath Learning Management System, a digital platform accessible on a smartphone app.

**Results:**

In total, 18 CHWs contributed to the adaptation of the SH+ content and digital intervention development. Participants commented on finding some terms difficult to follow and recommended simplifying the language and providing detailed explanations. Participants offered positive feedback on the adapted content, expressing that they found the examples covered in the material both relatable and relevant to their own personal experiences at home and in the workplace. By combining participants’ insights and comments with feedback from content experts, it was possible to finalize a digital Hindi version of the SH+ intervention for CHWs in rural India.

**Conclusions:**

This study is timely given the continued detrimental impacts of the COVID-19 pandemic, and offers a promising and potentially scalable digital program to alleviate psychological distress among frontline health workers.

## 1. Introduction

Community health workers (CHWs) represent the backbone of most health systems, responsible for delivering essential primary care services, particularly in remote and underserved settings in low- and middle-income countries (LMICs) (Vasan et al., 2017; Scott et al., 2018; Tulenko et al., 2013). CHWs are often members of the communities in which they serve (Pallas et al., 2013), whereby they can establish trust and are ideally positioned to deliver a range of services from preventive care and educating community members, to supporting ongoing management of chronic conditions, and acting as the essential connection between community settings and primary healthcare facilities (Black et al., 2017; Lewin et al., 2010; Perry et al., 2014). Despite serving a key role in most health systems, CHWs and other frontline providers take on significant workload and are at high risk of burnout (Dugani et al., 2018; Dubale et al., 2019; Wright et al., 2022).

In the context of India, ASHAs (i.e., Accredited Social Health Activists) represent the health worker cadre closest to the community that takes on an extensive range of programmatic responsibilities. ASHAs are women and community residents, with a minimum of 8 years of formal education, who act as volunteers while receiving incentive-based payments, and typically serve a population of about 1000 (Scott et al., 2019). The ASHA program was launched by the National Rural Health Mission (now National Health Mission), Government of India in 2005, primarily to improve maternal and child health outcomes, and has since broadened its ambit to strengthening the accessibility, availability and acceptability of a broader range of public health services in communities with a focus on enhancing service reach for poor and vulnerable sections (Agarwal et al., 2019; Scott et al., 2019). Over time, the roles and responsibilities of ASHAs have expanded to include conducting health surveys, improving reproductive and child health, and delivering preventive, promotive and curative services related to non-communicable disease programs, promoting vaccination, and supporting institutional births. Since its inception, the ASHA program has been instrumental in improving neonatal, child and maternal mortality, improving vaccination coverage and other key health outcomes across India (Scott et al., 2019). However, like other lower resource contexts, mounting evidence highlights the detrimental impacts of burnout and work-related stress among ASHAs in India, including poor motivation and reduced well-being (Pulagam and Satyanarayana, 2021; Manjunath et al., 2022), serious concerns that have been further exacerbated due to the COVID-19 pandemic (Niyati and Nelson Mandela, 2020; Mishra et al., 2022; Deng and Naslund, 2020). Specifically, recent studies from India have found that upwards of half of frontline health workers, including ASHAs, have experienced high risk of psychological distress during the pandemic, largely driven by longer work hours, emotional exhaustion, detrimental impact on sleep, and fears of contracting the virus (Yella and Dmello 2022; Menon et al., 2022).

ASHAs are particularly susceptible to facing work related stress and being overburdened, which are driven by unequal work distribution (Manjunath et al., 2022; Meena et al., 2020) as well as gender inequities within the health system (Ved et al., 2019). ASHAs experience both workplace and their own personal household stressors, which often makes it difficult for them to meet the multiple demands and expectations placed upon them (Aryal et al., 2019; Deshpande et al., 2019). At the health system level, prior studies have reported that key factors contributing to stress and burnout among ASHAs include low compensation and relying on incentives compared to other salaried health system personnel. Additional factors that contribute to burnout include delays in receiving outcome-based incentive payments, poor institutional support, perception of low incentives as compared to the nature and amount of work, unfulfilled training needs, and a dearth of support and adequate respect from other cadres of health workers (Scott and Shanker, 2010). ASHAs have also highlighted that due to being at the bottom of the health system hierarchy, they feel that they hardly have a ‘voice’ to express their concerns (Mishra, 2014). At the individual level, additional stressors can include patriarchal values embedded within the socio-cultural milieu, familial expectations around gendered role playing of domestic responsibilities, low socioeconomic status and coming from impoverished settings, and limited educational qualification (Ved et al., 2019; Manjunath et al., 2022). Therefore, ASHAs face a two-fold challenge of meeting ever-increasing expectations for addressing health issues of the communities they serve while simultaneously managing stressors experienced in their workplace and personal life.

The efforts needed to address these concerns will require major structural changes at the health system and societal levels. However, there may also be more immediate solutions aimed at promoting the mental health and well-being of frontline CHWs such as ASHAs. There has been a dearth of research addressing stress among CHWs in LMICs such as India (Dugani et al., 2018), while prior studies have predominantly focused on ‘extrinsic’ factors like monetary incentives, improved supervision strategies, flexible tasks, public recognition, and rewards, yet with inadequate consideration of the ‘intrinsic’ factors such as mental health and wellbeing of ASHAs themselves. Finding ways to support ASHAs in managing their stress and burnout could meaningfully support their wellbeing, and may also help them in their work roles and result in benefits for the patients and communities they serve (Willis--Shattuck et al., 2008; Mondal and Murhekar, 2018; Bajpai and Dholakia, 2011; Bhattacharyya et al., 2001; Singh et al., 2015). Therefore, there is a gap in the research on developing and testing an evidence-based, contextually adapted intervention for frontline workers like ASHAs, aimed specifically at strengthening their individual-level management of work-related stress and burnout.

Considering the complex factors contributing to compromised well-being of ASHAs, and drawing from the World Health Organization’s (WHO) five-pillared recommendations of protection of frontline health workers (World Health Organization, 2022), it becomes imperative to devise interventions to support ASHAs and facilitate change towards adopting strategies that can help them cope effectively (Otu et al., 2020; Bo et al., 2021). Recognizing that this would not address broader structural challenges, a brief scalable stress reduction program could offer immediate relief to a frontline workforce experiencing elevated burden and risk of burnout. The WHO has developed a culturally adaptable, low-intensity psychological intervention, called Self-Help Plus (SH+), for stress management (Epping-Jordan et al., 2016). Importantly, this brief intervention is transdiagnostic, and designed with the intention of being easily adaptable to different cultures and contexts while being beneficial and safe for individuals with and without mental disorders (Epping-Jordan et al., 2016). SH+ has demonstrated effectiveness in reducing stress among women in humanitarian settings in Uganda (Tol et al., 2018; Tol et al., 2018). In a cluster-randomized controlled trial (Brown et al., 2018) in 14 villages in Uganda enrolling 694 participants, SH+ contributed to significant reduction in psychological distress compared to enhanced usual care post-intervention (ES = −0.72) and at 3-months follow up (ES = −0.26) (Tol et al., 2020). Furthermore, in a randomized controlled trial conducted across 5 countries in Europe, when compared to treatment as usual, the SH+ intervention demonstrated effectiveness in preventing onset of mental disorders among refugees and asylum seekers post-intervention, though not at 6-months follow up (Purgato et al., 2021). Most recently, the SH+ intervention was found be effective in preventing mental disorders in a randomized controlled trial enrolling 642 Syrian refugees experiencing psychological distress in Turkey (Acarturk et al., 2022). Participants who received the SH+ intervention were significantly less likely to have any mental disorders at 6-month follow up compared to the usual care control group (22% vs. 41%, p < 0.001) (Acarturk et al., 2022).

SH+ has theoretical and conceptual roots grounded in Acceptance and Commitment Therapy (Levin et al., 2015; A-tjak et al., 2015), which can reduce symptoms of depression and anxiety (A-tjak et al., 2015), and combines elements of cognitive behavioral therapy including psycho-education, mindfulness exercises, and promoting psychological flexibility where individuals learn new ways to open up to and cope with difficult thoughts and feelings consistent with their own values, rather than avoiding these thoughts (Hayes et al., 2012). One key feature of SH+ is the broad focus on reducing psychological distress associated with adversity and difficult life experiences (Epping-Jordan et al., 2016), which increases scalability by not requiring use of diagnostic procedures or targeting specific syndromes. Specifically, the SH+ intervention endorses enhancement of psychological elasticity and has the following key components: grounding, unhooking from difficult thoughts and feelings, acting on values, being kind, and making room for difficult emotions. The original SH+ intervention is composed of multimedia and a manual, to be delivered in a group setting consisting of five weekly sessions. Each session focuses on a review of concepts and practice of skills from previous sessions, the introduction of new skills, brief group discussions and self-reflection exercises, and encouragement of daily practice of these key skills (Epping-Jordan et al., 2016). To our knowledge the SH+ intervention has not been previously adapted for use among CHWs in India, yet there is ongoing research using this program for addressing psychological stress among health workers in China (Luo et al., 2022). Furthermore, the simplicity of the program content and emphasis on skill building make this a potentially highly relevant program for empowering ASHAs to better cope with and manage a stressful work environment. Adapting the SH+ intervention to diverse settings also aligns with WHO efforts to develop a range of scalable and effective psychological interventions for release as public goods (Carswell et al., 2018; Harper et al., 2020; Purgato et al., 2019).

In our prior studies, we found that digital interventions are both feasible and acceptable for use with ASHAs in Madhya Pradesh (Muke et al. 2019, 2020), a finding that has similarly been demonstrated in other settings in India (Kapanee et al., 2022), as well as in settings with CHWs outside of India (Rahman et al., 2019). We have found that ASHAs have expressed keen interests in accessing content from mobile devices as it avoids the need to travel long distances and spend time away from their families to attend in-person programs (Muke et al., 2019). Furthermore, we have found in our ongoing work that nearly all ASHAs own cellphones, and over half own smartphones (Naslund et al., 2021), further highlighting the potential for leveraging these widely available devices to offer support and build resilience of this frontline workforce. Importantly, we have also observed high engagement in the use of digital programs accessible from a smartphone app (Muke et al., 2020), emphasizing that it may be an ideal approach for delivering a stress reduction program to this target group of health workers. Use of a mobile app can also avoid drawing attention beyond typical phone use (e. g., easily fits into daily activities), and could minimize concerns or feeling ashamed to seek help by delivering stress reduction content in a non-stigmatizing way (Naslund et al., 2019; Naslund and Deng, 2021). As smartphone ownership continues to increase, health systems in some states, such as Gujarat and Madhya Pradesh, are providing smartphones to frontline health workers including ASHAs to support them in their work (Modi et al., 2019; Saha et al., 2020), further highlighting the potential for a digital self-help intervention to offer widespread scalability. In addition to adapting the SH+ intervention for ASHAs, we also aimed to digitize the content and make it accessible in an individualized self-directed format, as opposed to the group-based format of the prior evaluation studies in Uganda, Turkey and Europe (Tol et al., 2020; Acarturk et al., 2022; Purgato et al., 2021). We intentionally selected the SH+ intervention for adaptation because it consists of many individual activities as well as an illustrated guide that could be adapted in such a way that it could be accessible from a smartphone app in a self-directed format, thereby avoiding the need for travel to a central location, and accommodating the busy schedule of ASHAs.

### 1.1. Study objectives

This paper describes the approach of adapting and digitizing the language, content and format of the evidence-based Self-Help+ (SH+) intervention for use with CHWs in rural Madhya Pradesh, India. The goal of our adaptation of SH+ was to ensure a culturally and contextually appropriate intervention accessible from a smartphone app for reducing psychological distress, and promoting the mental health and wellbeing of frontline CHWs in rural India. Importantly, we engaged CHWs in adapting and digitizing the evidence-based intervention content for use in India, drawing from the principles of human-centered design whereby the needs of the target user-group are considered central to the design process. Human-centered design can improve user satisfaction and relatability with the intervention, promote ownership of the program content, and enhance effectiveness and opportunities for uptake and sustainability (Wheelock et al., 2020; Triplett et al., 2021). This approach also builds on our prior successful efforts working with and engaging ASHAs in the development of digital training programs (Black et al., 2023). The present study represents the formative phase of the SAMBHAV (Stress Alleviation Methods for community-Based Health ActiVists) project, whose overarching aim is to develop and evaluate the feasibility, acceptability, and preliminary effectiveness of a digital self-directed version of the WHO’s SH+ intervention tailored to the needs, context, and local culture, and to explore the potential for reducing stress and promoting mental wellbeing among ASHAs in rural India.

## 2. Methods

### 2.1. Setting

This study was conducted in Sehore district, a rural district in the state of Madhya Pradesh, which is geographically one of the largest states of India with a population over 75 million, of whom nearly 73% reside in rural areas (Suryanarayana et al., 2016; Menon et al., 2008; Kokane et al., 2019; Shidhaye et al., 2017). This state lags behind many other Indian states and ranks among the lowest on the Human Development Index (Suryanarayana et al., 2011). This project expands on our research infrastructure in the region, and involves recruiting CHWs called ASHAs (Accredited Social Health Activists—deployed in India’s National Health Mission), and ASHA Facilitators, who provide monitoring and supervision for ASHAs (Scott et al., 2019). ASHAs were recruited from primary healthcare facilities in Sehore district to contribute to the development and adaptation of the digital SH+ intervention.

### 2.2. Research design

**Fig. 1.**
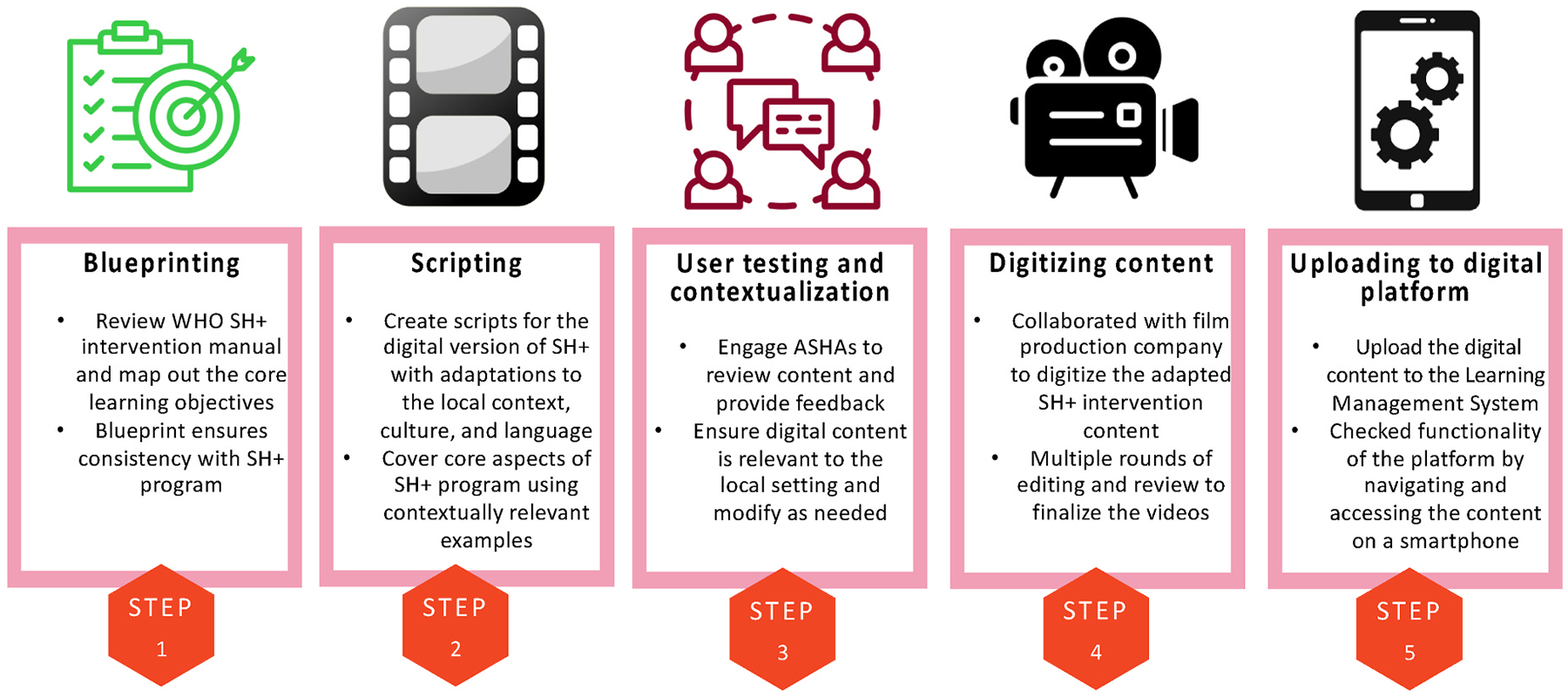
Overview of the stepwise approach to development of the digital program for stress reduction for community health workers in rural India.

We employed a stepwise design approach to guide the adaptation of the SH+ content and digital intervention development, informed by our prior work in developing interventions for the same target group of ASHAs in rural Madhya Pradesh (Khan et al., 2020) and grounded in principles of human-centered design (Black et al., 2023). Mainly, through engaging the target group of ASHAs throughout the design process to ensure that the adapted content is centered around their needs, which can enhance acceptability, user satisfaction, and improve chances for uptake and adoption (Baker et al., 2020). Additionally, the core content and the concept of the WHO SH+ program was followed throughout the adaptation process (World Health Organization, 2020). The WHO SH+ program covers the self-help stress reduction techniques and teaches the ways to deal with stress through simple exercises and methods. We ensured that the adapted content remained consistent with the original SH+ content by reviewing adapted materials with content experts and ensuring multiple levels of review within our own team. Given our earlier experience of designing and developing digital training interventions, drawing from prior literature in the development of digital interventions (Yardley et al., 2015), we followed a step-wise process to develop the digital self-help stress reduction program for ASHAs (Khan et al., 2020). The steps are illustrated in Fig. 1, and described in the sections that follow.

#### Step 1 Development of the digital intervention blueprint

First, we developed a blueprint for the digital stress reduction self-help program (Coderre et al., 2009), which involved carefully reviewing the original WHO SH+ intervention manual and mapping out the core learning objectives (World Health Organization, 2020). In the blueprint for the SH+ program, the different topics from the intervention manual, such as specific techniques and exercises required to deal with the stress and burnout, were organized into six modules, with each module further broken down into short segments covering key lessons. The blueprint serves as a roadmap to guide the development of the digital SH+ course content and acted as the architecture for the overall layout and of the digital self-care intervention. To ensure consistency with the original WHO SH+ intervention manual, the blueprint was created by one member of our research team, and then reviewed by three additional team members to double check that the modules outlined in the blueprint aligned with the original manual. Lastly, the blueprint was reviewed by an external researcher with expertise in the WHO program content. The final blueprint for the adapted version of the digital SH+ intervention is listed in Table 1.

#### Step 2 Development of SH+ program scripts and content

The development of the self-help content started with the adaptation of the written and graphical content of the WHO SH+ manual (World Health Organization, 2020). We considered our prior formative research activities and focus group discussions with ASHAs to support the development of script ideas that reflect personal stories that are relatable for ASHAs (Muke et al., 2019). The digital content described in the blueprint comprised images, graphics, and short videos illustrating the different skills and techniques covered in the program manual. Development of the video content for the program involved; first, writing scripts in English, following an approach that has previously been applied by our team (Khan et al., 2020). The scripts included original language of the WHO SH+ manual as well as additional supplemental content created to offer illustrative stories, scenarios and real-life examples that align with the local context and closely relate to the experiences encountered by ASHAs, and that could be interactive and engaging for the self-directed delivery format of the adapted digital version of the SH+ program. The scripting for the videos also involved describing use of local settings for filming, and included dialogues, montages and directions for shooting specifications. Two members of our research team (RS and SC) developed the scripts, and the drafts were further discussed within the team to reach consensus before circulating for review from additional research team members (AS, JRH, and JAN) and an external content expert (EK). We made further revisions to finalize the scripts by incorporating the feedback and comments from reviewers.

Since the target population of ASHAs in Madhya Pradesh are Hindi speaking, it was also necessary to translate the scripts. To ensure contextual fit, research team members from the same target setting completed the translation of the scripts, ensuring inclusion of appropriate terms and language reflective of the local culture and language. Other team members fluent in Hindi (RS, SC, and AS) then reviewed the translated scripts to check the alignment with the English content and to ensure simplification or removal of jargon or complex terms, given that many of our target group of health workers have 8th standard education and above, and may have only basic literacy levels. Our final step involved having a clinician with Master’s level training and extensive prior experience working with and training ASHAs in delivery of brief psychological interventions in the target setting in Madhya Pradesh review the Hindi versions of the scripts (AK). We completed a subsequent round of revisions of the Hindi scripts, ensuring that feedback from the multiple rounds of review was fully captured in the final scripts. The process of drafting the English scripts and then translation and finalizing the Hindi scripts was completed over approximately five months.

In parallel to the development of the scripts, our team drafted an adapted version of the WHO SH+ manual (World Health Organization, 2020), following a similar layout and format, but with culturally and contextually relevant images and graphics. Our team worked closely with a graphic designer to create specific images and icons to illustrate the different stress management techniques and strategies described in the SH+ manual. A member of our research team (RS) also carefully reviewed existing training and intervention materials designed for ASHAs and circulated by the Ministry of Health and Family Welfare in India to offer examples of context designed for the target group of health workers i.e., use of relatable and relevant language, scenarios, and examples in the content. Working closely with the graphic designer, our team provided feedback and comments on the different images and to ensure alignment with the original WHO SH+ manual content. After finalizing the images, the content was compiled into a manual format with additional text added (taken from the scripts). The SAMBHAV manual was developed in both English (to allow a version for translation into other languages in India) and Hindi.

#### Step 3 Content testing and contextualization workshop

This step involved engaging ASHAs to review the content and provide feedback on the cultural acceptance, relevant language modifications, stories and examples covered in the program. We invited 20 ASHAs, employing a convenience sampling approach by drawing from the larger pool of ASHAs enrolled in another ongoing research study (Naslund et al., 2021), to participate in a workshop with the objective to check the language of the content and the appropriateness of the stories included in the scripts, by collecting their perspectives and recommendations for improving the adapted SH+ program content. The participating ASHAs were aged ≥18 years of age, had minimum education level of 8th standard to ensure sufficient literacy level to operate the smartphone and to follow any of the written intervention content, and a willingness to stay in the study area over the duration of the study. Two members of our research team (RS and SC) facilitated the workshop over a 3-day period. On the first day, we tested module-1; followed by module-2 and module-3 in the second day; and module-4 and module-5 were saved for the last day. The ASHAs were informed about the program and invited for the content testing and contextualization workshop after securing necessary government approvals. Of the 20 ASHAs invited to join the design workshops, a total of 18 ASHAs participated, including one ASHA Facilitator. These ASHAs were all women, and ranged in age from 21 to 42 years, had between 8th standard to graduation level of education, and an average of seven years of work experience. Participating ASHAs were also provided with smartphones with the content pre-loaded to the mobile app to minimize potential technical challenges and to ensure access to the content for the purpose of feasibility testing.

The workshops were broken down into two segments. We first introduced the purpose to learn about any difficulties faced by the ASHAs in understanding the language used in the modules and any difficulty in understanding the content, terms, and relating the stories and roleplays to their real-life situations. Printed copies of the materials were given to the ASHAs to read, and they were asked to underline or encircle any sentences or words which they found difficult to understand or to mark any comments related to the stories. In the second session, after ASHAs completed the reading and commenting on the hard copies of the modules, members of our research team sat together with the participating ASHAs in two small groups and discussed the modules with them, and asked them about use of appropriate words, or suggestions for alternative words and sentences that could be used instead of the ones which they found difficult to read and understand. This was followed by a semi-structured focus group discussion to collect insights from this same group of participants from the workshops to inform further modifications and improvements to the content stories and roleplays in the modules. The focus group discussions were facilitated by two members of our team, and guided by targeted questions focused on different aspects of the digital program content, such as: “*Are these roleplays and stories relatable to your day-to-day and work-life situations*?” or “*Are you understanding the content? Could you describe any difficulties you experienced when reading the content*?” or “*Do you need more explanation or details regarding the content*?” and “*Any suggestion to use new words for the difficult words?*” Two members of our research team documented participants’ feedback and suggestions from the qualitative focus group discussions. Our analysis was guided by the need to inform improvements to the program content; therefore, we employed a rapid thematic analysis approach, consistent with prior studies focused on gathering health worker feedback for informing digital intervention development in low-resource contexts (Tyagi et al., 2023; Kumar et al., 2022), where the two researchers combined their observations, and organized participant feedback according to different aspects of the program that could be improved (i.e., language, stories, difficult wordings, etc.). Next, we convened a discussion with our larger investigative team to review the qualitative insights from participants, to reach consensus on what aspects of the content require improvements, and to plan out next steps for making these modifications accordingly.

#### Step 4 Digitization of the SH+ content

Our team collaborated with film production teams and actors to digitize the adapted SH+ intervention content. The videos were filmed in several locations, including community health centers, typical locations in villages, and the research office in Bhopal to show the culturally appropriate context in the digital content. The videos consisted of a combination of lectures delivered by a counsellor or fellow CHW, and the use of actors who could emote and deliver the lines, as well as role-play videos, where actors were selected to illustrate mock scenarios involving ASHAs learning and applying the skills and techniques covered in the SH+ intervention during routine everyday scenarios.

We completed the video shooting over 10 days. The research team members (RS and AS) were present during all the days of filming to guide and supervise video production, and to ensure that the filming adhered to the scripts covering the adapted WHO SH+ intervention content. The film production team was selected for this project as they had prior experience working with our team in the digitization of other program content, can ensure the production of high-quality video content, and because the ability for our team to work with them following the video shoot, allowing for multiple rounds of editing required to finalize the videos for uploading to the digital platform. A member of our team was also present throughout the editing process to review each iteration of edits to ensure the correct use of subtitles, icons and images, montages, placement of bullet points and text, sound quality for spoken content and narration, and use of music and other sound effects. Our team simultaneously worked to develop a series of PowerPoint videos consisting of graphics and with voice narration that would be used to supplement the video-based content in the program.

#### Step 5 Setting up the digital platform

Our team previously developed a Learning Management System (LMS) using the Moodle platform for hosting digital content. While the LMS software is typically designed for delivery of educational content, we opted for this same software for delivery of the SH+ intervention content given that we have previously demonstrated that this platform is both feasible and acceptable for use in the target population of ASHAs for accessing training content focused on depression care (Muke et al. 2019, 2020). Therefore, including targeted self-help content on this platform seemed likely an ideal approach for ensuring familiarity, uptake, and allowing self-directed interaction and use among our target group of ASHAs. Furthermore, given our experience working with this digital platform, uploading the digital content could ensure the timely development of a prototype ready for pilot testing. The structure of the SH+ course was created on the LMS and the various types of content, including videos, PowerPoint videos, graphics, and additional written content, was uploaded. Once the content was uploaded on the LMS, our team checked the functionality and the feasibility of the platform by navigating the platform and accessing the self-help digital content on their smartphones. We also ensured that the content was loaded directly onto smartphones so that it could be accessed in offline functionality to overcome potential technical challenges and to enable access for ASHAs in regions with low bandwidth. Two team members checked the navigation and feasibility, made any final corrections by primarily addressing any technical difficulties or minor changes for clarity in the content. Following this final round of internal revisions, the adapted digital SH+ program, called SAMBHAV (Stress Alleviation Methods for community-Based Health ActiVists), was ready for delivery to ASHAs and pilot testing to assess feasibility, acceptability, and preliminary effectiveness in alleviating stress in this target population of frontline health workers.

### 2.3. Summarizing adaptations to the SH+ intervention

Our development of the SAMBHAV intervention involved multiple types of adaptations to the SH+ intervention, including adapting the content for use in rural India, tailoring the materials for the needs of ASHAs, and digitizing the content to enable self-directed access from a smartphone app. Therefore, we provide a summary of the different types of adaptations organized according to an existing established model for cultural adaptation of psychological interventions, referred to as the ecological validity model (Bernal et al., 1995). As summarized in Table 2, this model outlines eight important dimensions of interventions, spanning language, persons, metaphors, content, concepts, goals, methods, and context (Bernal et al., 2009), and has been applied widely in the adaptation of brief psychological interventions for diverse settings (Perera et al., 2020; Mabunda et al., 2022). Documenting the different adaptations and modifications to the SH+ program content can enable replication, inform further adaptations by uncovering which dimensions of the program were sufficiently adapted or not, and support subsequent outcome evaluation (Naslund and Spagnolo, 2023).

### 2.4. Ethics

Ethics approval for this study was obtained from the Institutional Review Boards at Sangath, India (Number: AB_2021_73) and Harvard Medical School, USA (Number: IRB22–0974). All the study participants provided written informed consent to be included in the study. To ensure participants’ confidentiality, raw data were deidentified and maintained in password-protected computers.

## 3. Result

### 3.1. Key recommendations for informing digital intervention development

During the intervention development process, the 18 ASHAs joined the design workshop and focus group discussions at our site office in Sehore district. Importantly, they offered feedback and recommendations pertaining to the content at the early stages of the digitizing process. In general, the ASHAs liked the content, and they perceived it to be new to them initially, especially considering some of the concepts and words that were introduced pertaining to identifying and managing stressors. Then, after having a chance to read through the content and examples in greater details, as well as review stories and images in the scripts, it was observed that the ASHAs found the content to be understandable and easy to follow. They only provided feedback regarding some of the technical words that were used, such as “hooking”, “grounding”, or “bridge” in the context of stress reduction. Most of the participating ASHAs highlighted these terms as being difficult to understand and suggested using simpler words and offering additional details and explanation of technical words. Regarding the roleplays and stories created for the training content, the ASHAs reported that they liked the explanation of content throughout the roleplays and stories, and they expressed being able to relate to these to their own real-life situations as well as their experiences of workplace stress. Table 2 provides a summary of the specific adaptations to the SH+ intervention content according to the eight dimensions of the ecological validity model proposed by Bernal et al. (1995).

### 3.2. Digital SH+ program called SAMBHAV

After integration of the feedback and recommendations of the participating ASHAs, the result of this project was the development of SAMBHAV, a fully digital SH + intervention package accessible from the Sangath LMS on a smartphone app. The adaptation and development of the digital SH + intervention was completed over 12 months from August 2021 to September 2022. The final digital program consisted of 6 modules, as summarized in Table 3. These modules contained a total of 20 short videos with 3 video lectures, 8 role-play videos, 6 PowerPoint lectures with voiceover, and 3 summary videos providing a recap of the content displayed in a given module. The videos ranged in duration from about 1 min to 13 min, with the average duration being approximately 6 min. We specifically kept the videos in short segments based on user feedback because this made it easier to stay engaged with shorter videos when accessing the content from a smartphone app (while also making it easier to load the digital content when considering challenges with low-bandwidth). In addition to the video content, we included a total of 27 engagement quizzes and 30 knowledge assessment questions embedded throughout the modules. The total duration of the digital SH+ intervention was about 4–5 h of delf-directed content.

**Fig. 2.**
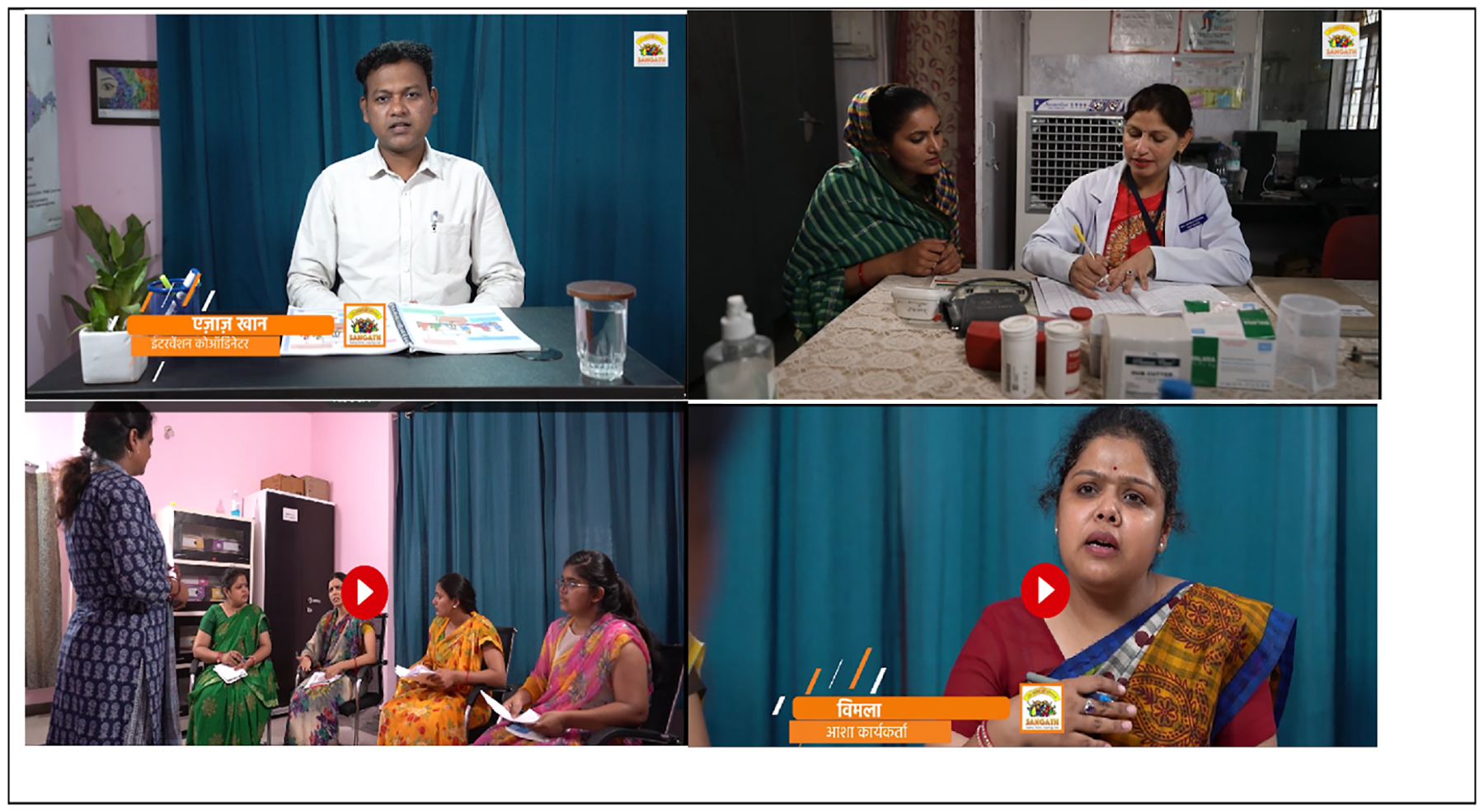
Sample video content from the digital SH+ intervention adapted for ASHAs in rural India.

**Fig. 3.**
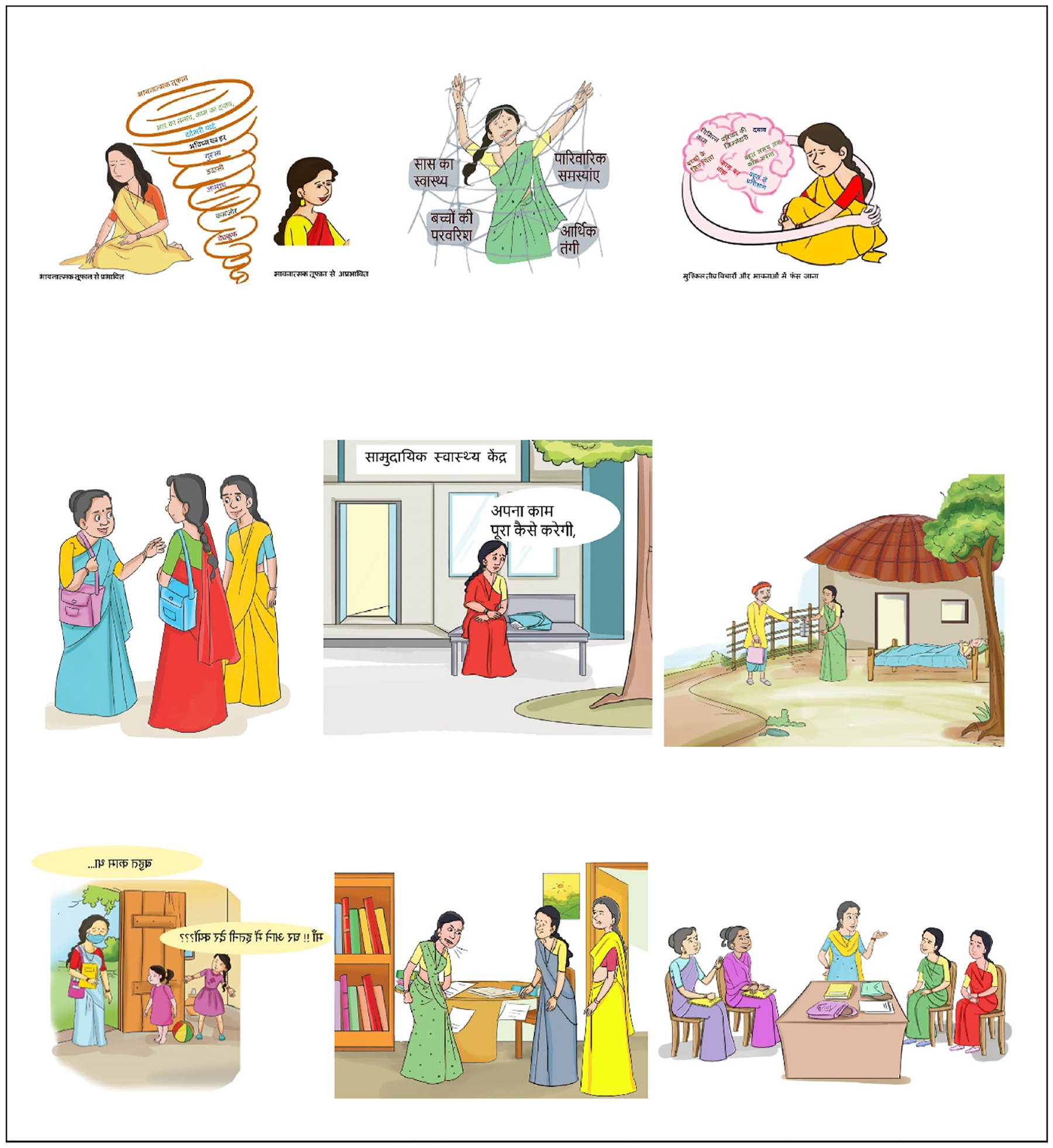
Sample graphics from the digital SH+ intervention adapted for ASHAs in rural India.

As illustrated in Fig. 2, the central components of SAMBHAV, i.e., the adapted digital SH+ intervention, were the instructional videos. These short videos consisted of lectures covering the core content of SH+, as well as role play videos demonstrating the application of the various skills and techniques for managing stress in everyday scenarios tailored to what ASHAs may typically encounter. The lectures included a variety of digital elements to support learning, such as visual graphics, text in bullet points, figures, tables, narration, footage from local community settings and health facilities, and example interactions between patients and health care providers in the community or at the clinic. Role-play videos were used to demonstrate the use of specific self-help techniques, and interaction between ASHAs and counsellors during learning sessions. The lecture and role-play videos were supplemented with PowerPoint lectures with voiceover, and with use of graphics and images tailored to the local context (see sample graphics in Fig. 3). We also used a variety of engagement and assessment activities, such as multiple-choice questions, drag and drop response options, and true/false questions covering the content to accompany the instructional videos and to support participant engagement in the program. The final digital content was uploaded to the Sangath LMS and is accessible from a smartphone app.

### 3.3. Illustrated SAMBHAV manual

**Fig. 4.**
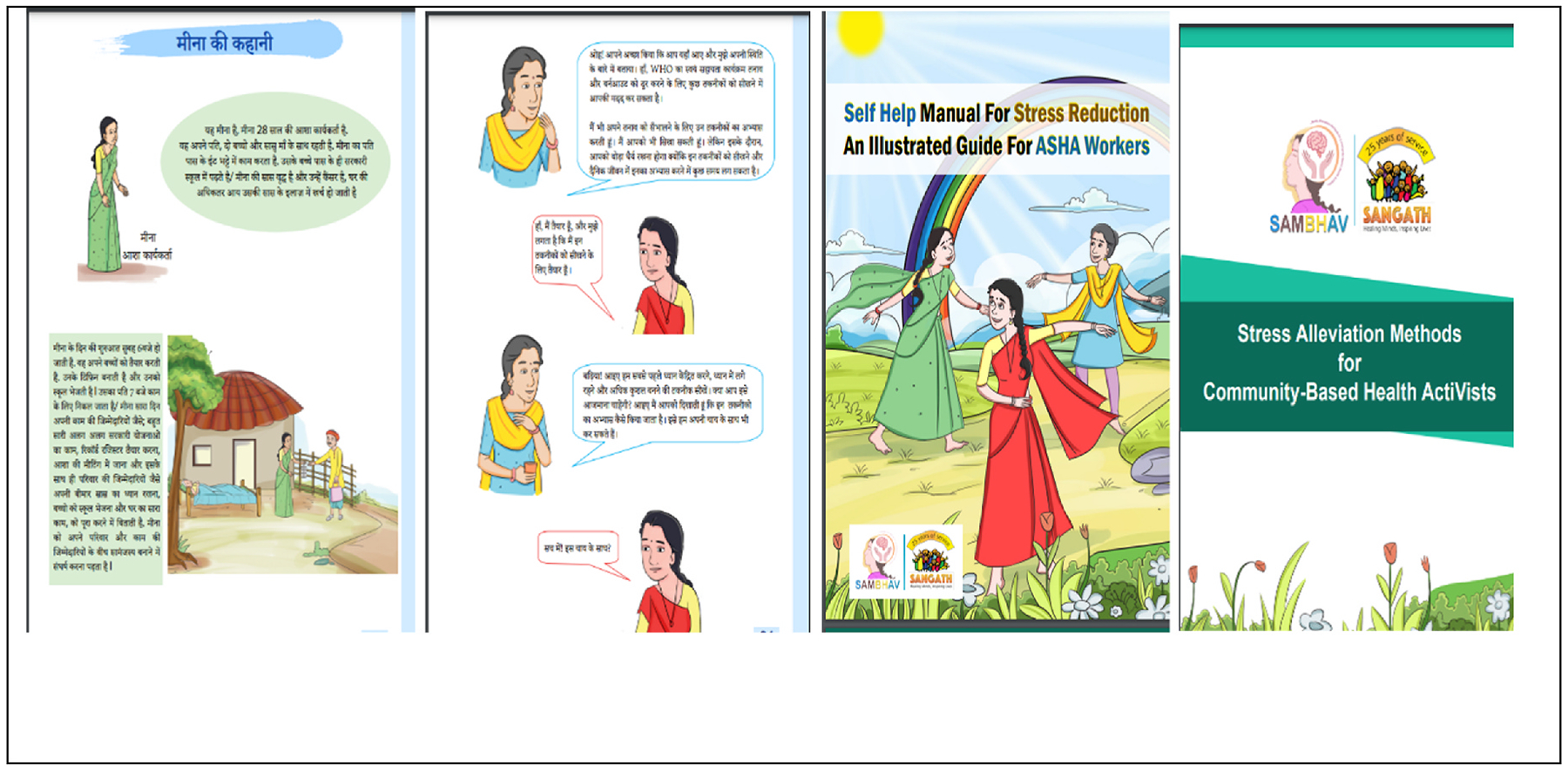
Sample content from the SAMBHAV program manual adapted from the WHO SH+ manual for use with ASHAs in rural India.

The SAMBHAV manual was designed to specifically include storytelling and relatable examples for ASHAs, and is closely aligned with the original WHO SH+ manual content and layout. The manual follows the same six modules in the digital intervention, offering a supplement that participants can use to reinforce the concepts and revisit the different stress management techniques and strategies if the digital platform is not available or to allow flexibility for accessing a different format (for instance in areas with low-bandwidth). In each module there are different characters, such as ASHAs and a counsellor, who are narrating their own stories of experiencing low mood and burnout and the solutions that they employed to overcome these challenges as outlined in the SH+ intervention. The characters and the stories were inspired from the real-life incidents and the problems that ASHAs face in their daily working life and in maintaining work life balance. Sample content from the illustrated manual is presented in Fig. 4.

## 4. Discussion

This preliminary study involved the development and adaptation of a digital version of the WHO SH+ intervention consisting of multiple rounds of review within our research team, and integrating feedback from ASHAs to ensure that the content was systematically adapted to the local language and culture, while remaining consistent with the evidence-based SH+ program materials (Epping-Jordan et al., 2016). Importantly, this study builds on our team’s track record developing digital programs for training and supporting ASHAs in rural India (Muke et al. 2019, 2020; Joshi et al., 2022; Khan et al. 2020, 2023; Tyagi et al., 2023), as well as our established research infrastructure in the region (Shidhaye et al., 2019), and longstanding collaboration with the Ministry of Health and Family Welfare, Government of Madhya Pradesh. The digital adapted SH+ intervention for ASHAs developed in this study, called SAMBHAV, holds promise for offering a scalable self-directed approach for reducing stress to support frontline community health workers in primary care settings in India.

This study adds to mounting recognition of the need for novel approaches to address the elevated burnout and distress impacting front-line health workers in LMICs (Wright et al., 2022). CHWs in LMICs are at an increased risk of burnout and distress and many of these frontline health workers live in the same impoverished communities as the patients they serve, travel long distances to rural and underserved areas, and receive low compensation for demanding and emotionally exhausting work (Li et al., 2014; Haq et al., 2008; Selamu et al. 2017, 2019; Dugani et al., 2018; Saprii et al., 2015). Though multiple research studies in LMICs have acknowledged that higher levels of distress and risk of burnout among frontline providers are a result of heavy workload, lack of perceived support, gender inequality, health system power hierarchy, interpersonal conflict, younger age, fewer years in practice, and lower work satisfaction (Ge et al., 2011; Malakouti et al., 2011), there remains a scarcity of research studies focusing on implementing support programs for the well-being of these frontline health workers. Studies have also found that CHWs report being more likely to quit their job when facing these higher levels of burnout (Kim et al., 2018; Mbanga et al., 2018). Despite recent global health system strengthening initiatives focused on enhancing performance of CHWs to improve patient outcomes, increase access to care, and adhere to treatment protocols (Ballard and Paul, 2017; Vasan et al., 2017; Agarwal et al., 2019), there has been virtually no consideration of the mental health needs of this workforce (Naimoli et al., 2014; Scott et al., 2018; Heller et al., 2019). Therefore, addressing psychological distress among CHWs, such as through mindfulness-based skills training (Jacobs et al., 2017) or using evidence-based self-help stress management techniques (Epping-Jordan et al., 2016), could strengthen workforce capacity and overcome challenges to sustained delivery of quality care (Dugani et al., 2018).

Due to the impacts of the COVID-19 pandemic the current study is timely as there have been a multitude of reports documenting the significant increase in workload and resulting risk of burnout among CHWs like ASHAs (Deng and Naslund, 2020). Among ASHAs, recent studies during the pandemic have documented that there has been an ‘intensification’ in their daily responsibilities and tasks, as reflected by an increase in as many as 2–3 additional work hours per day given the added demands for responding to the pandemic containment and vaccination efforts (Niyati and Nelson Mandela, 2020; Upadhyaya, 2022). Additionally, ASHAs have reported receiving reduced or irregular pay given that many incentive-based activities were suspended, or delayed payments, while simultaneously being required to work in unsafe conditions without adequate protective equipment (Niyati and Nelson Mandela, 2020; Nanda et al., 2020; Upadhyaya, 2022). In the most serious instances, ASHAs experienced elevated mental distress as they were the target of discrimination and violence in several communities given their role in containing the pandemic and facilitating case finding activities, further fueled by fears of contracting the virus as well as fears among villagers that they could spread the virus (Konduru et al., 2022; Upadhyaya, 2022). With recent research and reports shedding light on the many challenges that ASHAs have been facing in their day-to-day work over the course of the COVID-19 pandemic, there have been calls to improve the working conditions and ensuring dedicated efforts to both empower and address the psychosocial and material needs of this essential workforce (Nanda et al., 2020). Specifically, the scalable digital stress reduction program developed in this study could complement additional efforts aimed at providing intrinsic support to ASHAs, and potentially mitigating some of the lasting consequences of the pandemic.

Our study also expands on new efforts seeking to leverage widely available digital technologies for supporting frontline health workers in diverse settings across India. While many of these prior studies, including work by our team, have primarily focused on training and supervision for ASHAs (Saha et al., 2020; Singh et al., 2021; Gopalakrishnan et al., 2020; Muke et al., 2020; Kaphle et al., 2016), few programs have considered whether digital technology could enable delivery of brief self-help interventions aimed at empowering ASHAs in responding to work-related stressors. Our study advances these efforts by considering the potential to leverage these same digital platforms used for training to offer stress reduction content aimed at preventing burnout and promoting the wellbeing of the workforce. This also is complementary to recent developments in India, where the disruption to routine care services caused by the COVID-19 pandemic has resulted in accelerated adoption and use of digital technology throughout the health system (Narayanan, 2021). This has also sparked the Government’s launch of the National Digital Health Mission as part of the Ayushman Bharat initiative in 2021, with the aim to develop the backbone necessary for supporting an integrated digital health infrastructure across the country (National Health Authority, 2022). As a result, there has been renewed interest in the use of digital technologies for supporting non-communicable disease care (Gudi et al., 2021), and specifically for training and supporting frontline health workers (Gautam et al., 2021). Therefore, there is immediate potential and opportunity for digital technology to achieve wide reach for supporting this essential workforce (Ved et al., 2019).

### 4.1. Limitations

It is important to consider some of the limitations with this initial formative study. While we describe the process for adapting the WHO SH+ intervention content and then developing the digital intervention tailored to the context for ASHAs in rural India, we have not yet assessed whether the digital SH+ intervention is effective and can achieve its desired goal of reducing psychological distress and burnout, and promoting mental health and well-being of the target group of ASHAs. Furthermore, continued efforts will be needed to determine whether such a scalable digital intervention, when made available across the broader workforce of ASHAs, can contribute to improvements in their job satisfaction, work performance, and ultimately, improving patient care while preventing health worker burnout and attrition. It seems intuitive that mentally healthy and motivated ASHAs will be better positioned to deliver high quality care to their patients; however, implementation research is necessary to consider this potential relationship, particularly in lower resource settings like India. As our team systematically adapted the evidence-based WHO SH+ intervention content for use with ASHAs in rural India, and further digitized the content for self-directed delivery on a smartphone application, it is possible that additional perspectives related to the feasibility and acceptability of the intervention content were not sufficiently captured given the small sample size. Furthermore, there was limited involvement of ASHA Facilitators in this study, which could partly be because the ASHA Facilitators were too busy as they typically assume a greater workload relative to ASHAs. This highlights the need to reach this group as they could stand to benefit from access to the digital stress reduction intervention. We also recognize that the ASHAs who enrolled in this study came from one rural district in a large state in Central India, and that they were previously engaged in other research and training activities being conducted by our team. It is likely that they were already highly interested in the focus of the current study and had prior exposure to digital intervention content, and therefore may not be representative of ASHAs in other settings across India. We worked to overcome this concern by tailoring the content to the everyday experiences of ASHAs, while simultaneously maintaining consistency with the original content of the SH+ intervention manual to ensure widespread applicability. Nonetheless, further region-specific modifications may be necessary to accommodate diverse languages, cultures, and contexts. Additionally, we focused specifically on reaching ASHAs with the adapted SH+ intervention because ASHAs represent the largest single workforce of CHWs on the globe, numbering over 1 million (Ved et al., 2019); however, ASHAs are all women, meaning that our findings likely do not generalize to male frontline health workers, highlighting another important focus area for future efforts. While use of digital interventions hold potential for achieving greater scalability, the reliance on smartphone devices with adequate connectivity may create barriers for reaching ASHAs working in the most impoverished settings where there is limited bandwidth, while also limiting the use of such interventions for CHWs who do not have access to digital devices or who may not have the necessary digital literacy skills for navigating the program content. In India, there has been increasing adoption of digital technology among frontline health workers in recent years (Bashingwa et al., 2021), yet ongoing attention will be necessary to overcome digital barriers to achieving scale up of such programs. For example, one approach could involve use of the written content for the program augmented with brief SMS text messaging support from a health coach to avoid the need for more costly smartphones and reliable online connectivity. Lastly, the current study and intervention development process was voluntary, meaning that the ASHAs who chose to contribute to our work may have already been experiencing distress, and therefore, may have been especially interested in learning more about stress management techniques. This may limit the generalizability of our program, highlighting the need for real world testing where the program can be made more widely available and allow tracking of levels of engagement and adoption among the target population of ASHAs.

## 5. Conclusion and next steps

The COVID-19 pandemic has put additional stress and risk of burnout on healthcare providers in resource constrained settings, and especially among frontline CHWs such as AHSAs in rural India. The need of the hour is to care for the carers and provide psychosocial support promoting the mental health and well-being is one of the key recommendations offered by the WHO for ensuring an efficient and responsive healthcare system (World Health Organization, 2022). There has been limited consideration within most health systems globally on the mental health and psychosocial needs of CHWs (Wright et al., 2022); therefore, the SAMBHAV program adapted from the WHO’s SH+ intervention holds promise. Our immediate next step is to pilot test the final digital intervention developed in the current formative research study with a larger cohort of ASHAs, where we will have the opportunity to collect measures of preliminary effectiveness in reducing psychological distress, while also capturing additional insights related to feasibility and acceptability of the program that could inform further modifications to facilitate implementation and scale up. If found to be effective, we are confident that the SAMBHAV digital stress reduction program would help to address an immense unmet need by empowering frontline community health workers to recognize work-related stressors and equip them with skills for managing these challenges and negotiating other concerns in their daily work and life.

This formative study is the first of its kind in India, and offers an initial step towards promoting the mental health and well-being of the world’s largest cohort of CHWs, the ASHAs numbering over 1 million, using a scalable digital version of the evidence-based WHO SH+ intervention (Tol et al., 2020; Epping-Jordan et al., 2016). Our preliminary efforts also set the groundwork to be considered by the state and central health systems policymakers to adapt and adopt this simple digital intervention for improving the well-being of their critically important work cadre. These efforts can also extend beyond the study sites in India for supporting CHWs in other low-resource settings, especially in the context of strengthening health systems due to the impacts of the COVID-19 pandemic. By recognizing the mental health and well-being needs of CHWs such as ASHAs, our exploratory study addresses a major gap in the global health literature, where there is urgent need to address the elevated stress and risk of burnout experienced by these frontline health workers (Wright et al., 2022).

## Figures and Tables

**Table 1:** Blueprint for the digital Self-Help+ intervention adapted for ASHAs in rural India.

Module No.	Module	Learning objectives	Learning outcomes	No of Chapters/Videos per Module (Total videos =)
1	Grounding	Learning about stress and its causes To know how stress impacts your body and mind Learning about stress management To know about a term hooking Learning about how to be more focused and engaged Practising Engaging and Focusing Exercises Knowing about simple tasks to perform with Family and friends can help overcome hooking situations To know about emotional storm. To learn and practice grounding exercises	After this module the learner acquires the knowledge: About stress and it differs About how stress impacts one’s body and mind About the concept of ‘hooking’ About the ways to be focused and engaged in day-to-day life About the skills of ‘engagement and focus’ and is ready for practising these About the simple tasks helpful in overcoming ‘hooking’ situations About what is an emotional storm Help you to engage, focus and unhook from difficult thoughts and feelings About how grounding exercises can be practised	**Video-1:** Stress, its causes and effects **Video-2:** What does hooked mean? **Video-3**: What is Focus, Engage and Pay attention and how does it help? **Video-4:** Awareness of drinking exercise **Video-5:** Grounding exercise-1 & 2 (or separate video on exercise-2)
2	Unhooking	To learn about unhooking ourselves from difficult thoughts and feelings To know about steps of unhooking To learn and practising notice and name exercises.	After completing this module, the participant(s) will be: Able to understand the ways to unhook from unwanted thoughts and feelings To be able to know the detailed steps of ‘unhooking’ Able to use and practice Noticing and Naming techniques.	**Video-1**: Learn unhooking **Video-2:** Notice and name exercise **Video-3:** Toolkit to practice grounding and unhooking
3	Acting On Your Values	To develop a detailed understanding of specific ‘Values’ To know the difference between Goals and Values To know about action plans and how to put your values into actions	After this module the participant(s) will be: Equipped with the knowledge about the most important values to them Able to differentiate between goals and values Able to develop a plan and put their values into actions	**Video-1:** Values **Video-2:** Action plan **Video-3**: Values and action plan exercise
4	Being Kind	To learn about how to be kind Learn and practice unhooking from unkind thoughts exercise	After completing this module, the participant(s) will be able to: Know how to be kind to themselves in contrasting situations Have an understanding of the harmful effects of being hooked to unkind thoughts Develop a plan and practice unhooking themselves from unkind thoughts	**Video-1:** What is being kind means? **Video-2:** Unhooking from unkind thoughts exercise
5	Making Room	To learn how to make a room for problems Learn and practice how to make room To learn about to be kind and practice being-kind exercises	After completing this module, the participant(s) will be able to: Gain knowledge and skills of making rooms for problems Practice the skills of being kind	**Video-1:** What is making room **Video-2:** Making room exercise **Video-3:** Being kind to yourself exercise
6	Tool Exercises/new exercises with examples	To revise the summary of grounding and practice its exercises To revise and summarize acting on your values and practice its exercise To summarize and practice being kind exercise To summarize and practice making room exercise	After completing this module, the participant(s) will be able to summarize and practice key concepts and exercises related to: Grounding including unhooking Acting on values Being kind Making room	**Video-1:** Grounding, unhooking **Video-2:** Acting on your values, being kind **Video-3:** Making room

**Table 2:** Summary of adaptations to the SH+ intervention.

Intervention dimension	Description of adaptation principle	Summary of adaptations to the SH + intervention
Language	Translation of the program content to the local language; use of culturally appropriate terms	Content was translated into Hindi and use of local terms was included Simplification of the language to match literacy levels of the ASHAs
People	Role of ethnic/racial similarities and differences between persons depicted in the program content and target population	Graphics throughout the digital content and written content depicts individuals from the local setting in rural India Use of vignettes with characters that are relatable and familiar to the ASHAs
Metaphors	Use of symbols and concepts that are familiar with the target population	Inclusion of real world examples of stress and burnout from the stories shared by ASHAs Illustrating the stress management techniques using imagery and examples that are familiar to ASHAs
Content	Alignment between the program content and the cultural knowledge, values, and local practices of the target population	Content was filmed in local settings, such as villages, communities or clinics, to ensure relevance for ASHAs The stories depicting examples of distress in the workplace were based on examples shared by ASHAs
Concepts	Ensuring the program content aligns with concepts of stress that are familiar to the target population	Many of the terms from the SH + intervention were adapted to ensure understanding and relevance for ASHAs The stress management techniques were tailored to align with activities that are familiar for ASHAs
Goals	Program goals align with social and cultural values of the target population	Goal of the adapted content was focused on identifying and responding to stress in work and home for ASHAs Emphasis placed on recognizing stress and empowering individuals with the skills to respond to stress
Methods	Program delivery in a format that is acceptable to the target population	Content was digitized to allow convenient access for ASHAs The program was adapted for self-directed delivery to accommodate the busy schedules of ASHAs and to avoid creating additional burden
Context	Consideration of barriers to accessing and benefitting from the program content for the target population	A written manual was created to follow the digital content as some ASHAs expressed interest in written content The written content can also overcome potential challenges with digital access

**Table 3:** Summary of digital SH+ intervention content in final adapted program.

Module	Type of Video	Duration of Video (In Minutes)
**Module-1**		
Video 1. Stress, its causes and effects	Lecture	8.51
Video 2. What does Hooked mean?	Lecture	10.21
Video 3. What is Focus, Engage and Pay attention and how does it help?	Lecture	2.11
Video 4. Awareness of drinking exercise	Role play 1	7.00
Video 5. Grounding exercise 1	Role play 2	10.12
Video 6. Grounding exercise 2	Role play 3	2.53
**Module-2**		
Video 1. Learn Unhooking	Role play 4	3.00
Video 2. Notice and Name Exercise	Role play 5	6.10
Video 3. Toolkit to practice Grounding and Unhooking	PPT-video	3.00
**Module-3**		
Video 1. Values	PPT-video	4.00
Video 2. Action Plan	PPT-video	5.15
Video 3. Values and action Plan Exercise	Role play 6	5.52
**Module-4**		
Video 1. What is being kind means?	PPT-video	10.11
Video 2. Unhooking from unkind thoughts Exercise	Role play 7	6.15
**Module-5**		
Video 1. What is Making Room	PPT-video	4.20
Video 2. Making Room Exercise	Role play 8	11
Video 3. Being Kind to Yourself Exercise	Role play 9	8.04
**Module -6**		
Video 1. Summary of Module 1	PPT-video	9.00
Video 2. Summary of Module 2 & 3	PPT-video	6.00
Video 3. Summary of Module 4 & 5	PPT-video	5.35
